# Berberine protects against lung injury induced by liver transplantation through upregulating PPARγ and suppressing NF-κB-mediated pyroptosis pathway

**DOI:** 10.1590/acb404925

**Published:** 2025-07-25

**Authors:** Wenna Liu, Xiaohui Liang, Mingxia Huo, Yongwang Wang, Guanghua Zhang

**Affiliations:** 1 Tianjin Medical University – I Memorial Hospital-Department of Anesthesiology – NHC Key Laboratory of Hormones and Development – Tianjin – China; 2 Tianjin Institute of Endocrinology – Tianjin – China; 3 Tianjin Medical University – Department of Pathology – Tianjin – China.; 4 The First Affiliated Hospital of Guilin Medical University – Department of Anesthesiology – Guangxi Province – China.

**Keywords:** Liver Transplantation, Lung Injury, Pyroptosis

## Abstract

**Purpose:**

We builted a orthotopic autologous liver transplantation (OALT) model in rats to evaluate the possible mechanisms of berberine against lung injury.

**Methods:**

Forty clean grade Sprague-Dawley rats (male, healthy, 250–280 g) were divided into five groups (n = 8): sham-operated group (group S), orthotopic autologous liver transplantation group (group T), berberine group (group B), peroxisome proliferator-activated receptor gamma (PPARγ) inhibitor GW9662 group (group G), and berberine + GW9662 group (group B+G). In group S, the relevant tissues around the liver were dissociated only. Orthotopic autologous liver transplantation was used in other groups, berberine 200 mg/kg/day was given one week before surgery in group B and group B+G. GW9662 1 mg/kg was intraperitoneally injected in group G and group B+G 4 hours before surgery. Blood samples were obtained for detecting PaO_2_ and the concentration of serum clam cell protein (CC16), surfactant protein-D (SP-D), interleukin (IL)-1β and IL-18. The immunohistochemical method detects the expression of PPARγ and nuclear factor-kappa B (NF-κB) in lung tissues. The expression of PPARγ, NF-κBand pyroptosis-related proteins were analysed by western blotting.

**Results:**

Rats exhibited increased histological lung injury following OALT. Liver transplantation caused upregulated CC16, SP-D, IL-18 and IL-1β levels, reduced PaO_2_ and the PPARγ expression, upregulated the NF-κB and pyroptosis-related protein expressions. BBR pretreatment greatly alleviates these lung damages induced by OALT. However, administration of GW9662 partially reversed the beneficial effects of BBR on lung injury.

**Conclusion:**

Berberine may play protective capacities against lung injury by upregulating PPARγ to downregulate the NF-κB-mediated pyroptosis pathway.

## Introduction

Up to now, liver transplantation is the best treatment for severe liver diseases, such as cirrhosis and acute liver failure. During liver transplantation, the portal vein is blocked, intestinal blood return is blocked, intestinal permeability is increased, and bacterial flora is translocation. When blood flow is reopened in the new liver stage, acidic metabolites and cryogenic perfusion fluids enter the systemic circulation, causing systemic inflammation responses, and the lung is the earliest organ to be damaged^1^. Acute lung injury can affect the recovery of liver transplantation patients and leads to the death of the recipient after liver transplantation.

Many studies have confirmed that early pulmonary complications generally included pneumonia, pleural effusion, atelectasis, and pulmonary edema, and the incidence rate ranged from 34.2 to 77.8%, which seriously affecting graft function and patient prognosis^2^,^3^. Early detection and timely management of pulmonary complications can improve the success rate of liver transplantation and quality of life of patients.

Pyroptosis is a mode of inflammatory cell death discovered in recent years and is a common innate immune mechanism in vertebrates^4^. Different from apoptosis and necrotic cell death, pyroptosis often occurs in conjunction with inflammatory response and NLRP3 inflammasome. Activating the NLRP3 inflammasome is a classic pathway and a key link to pyroptosis. More and more evidence shows that pyroptosis is involved in organ injury. A study has found that buformin improves acute lung injury caused by sepsis, possibly through inhibiting NLRP3-mediated pyroptosis^5^. Hao et al.^6^ showed that pyroptosis is involved in fatty liver injury. Nicorandil is a good treatment drug for ischemic heart disease, which can alleviate pyroptosis caused by myocardial infarction and play a cardioprotective role^7^. We hypothesized that pyroptosis may be associated with the inflammatory reactive of lung injury after liver transplantation.

Berberine (BBR) is a kind of alkaloid extracted and isolated from Chinese herbal plants, which has little toxic side effects and has been used to treat many diseases in China^8^. It has anti-inflammation, anti-cancer, and other biological activities^9^,^10^. Some studies have found that BBR can improve lung injury induced by multiple factors. BBR can improve acute lung injury through downregulating nuclear factor-kappa B (NF-κB) related pathway in septic mice^11^. Chen et al.^12^ confirmed that BBR can inhibit inflammation response in lipopolysaccharide rat model via the NF-κB/Nlrp3 pathway. Additionally, BBR provided an effective treatment for lung injury by suppressing leukocyte adherence in lipopolysaccharide-stimulated vascular endothelial cells^13^.

Our main purpose in this study was to research the possible beneficial mechanisms of BBR on lung injury during liver transplantation.

## Methods

### Animals

Forty Sprague-Dawley rats (male, healthy, 250–280 g; Spiff Biotechnology Co., Beijing, China) were housed in the Laboratory Animal Center of Tianjin Medical University Chu Hsien-I Memorial Hospital under a controlled conditions–20–26°C, 40–70% humidity, 12-h light/dark cycle (7 a.m.–7 p.m.). Animals were accessed to drink freely but fast for 8 hours before surgery.

The study was approved by Experimental Animal Ethics Committee of Tianjin Medical University Chu Hsien-I Memorial Hospital (DXBYY-IACUC-2020037). The rats with strong physique, free movement and keen response to external stimuli were selected for the experiment.

### Groups

The animals were randomly allocated into five groups:

- Group S: sham-operated group (n = 8);
- Group T: orthotopic autologous liver transplantation group (OALT group, n = 8);
- Group B: pretreated with berberine (200 mg/kg/d, intragastric administration) for one week+OALT (berberine+OALT group, n = 8);
- Group G: pretreated with peroxisome proliferator-activated receptor gamma (PPARγ) inhibitor GW9662 (1 mg/kg, intraperitoneally) for 4 hours+OALT (GW9662+OALT group, n = 8);
- Group B+G: pretreated with berberine (200 mg/kg/d, intragastric administration) for one week + pretreated with GW9662 (1 mg/kg, intraperitoneally) for 4 hours before surgery+OALT (berberine+GW9662+OALT group, n = 8).

In the group S, the abdomen was only opened, the blood vessels and ligaments around the liver were freed, and the abdomen was closed without vessel occlusion. Rat models of orthotopic autologous liver transplantation was prepared in all other groups except group S.

### Experimental animal model

After giving an abdominal injection of anesthesia with 2.5% pentobarbital sodium (50 mg/kg), abdominal hair of the rats was depilated, and the skin was prepared. Iodophor was used for disinfection. The falcate and the left triangle ligament of liver were firstly cut. Then, the suprahepatic vena cava (SHVC) and the infrahepatic vena cava (IHVC) were carefully separated and fully exposed. The hepatoduodenal ligament was cut open, and the portal vein (PV) and proper hepatic artery were separated. PV, proper hepatic artery, and IHVC were blocked with a vascular clamp. A 1-mL insulin infusion needle containing 30 U heparin saline was punctured above the PV block parts, making liver blood flow into the systemic circulation. The whole liver became ischemic during this process. Then, SHVC was blocked with a vascular clamp.

The anhepatic phase began. 4°C sodium lactate ringer’s solution was perfused continuously, and a small opening in the IHVC was cut as outflow tract. The time of the entire ischemic time was 30 min. This procedure was used to simulate the cold preservation of the liver during liver transplantation. It can be seen that the liver after perfusion gradually turned earthen yellow. Finally, the IHVC outflow tract and PV puncture site were sutured with 9-0 non-injurious vascular lines. SHVC, IHVC, PV, and proper hepatic artery were opened successively to restore blood circulation of the liver. Reperfusion of the liver was induced. The abdominal cavity was irrigated with warm normal saline and closed. Rats were anesthetized again 6 hours after reperfusion. The abdominal aortic blood and lung tissues were harvested for further analysis.

### Pulmonary oxygenation function

Abdominal aortic blood at 6 hours after autologous orthotopic liver transplantation was detected using a ABL90 FLEX automatic blood gas analyzer to measure the arterial oxygen tension (PaO_2_) level. This result was used to evaluate the gas exchange function of lung tissues.

### Histological examination

The collected lung tissues were fixed in 4% paraformaldehyde for at least 24 hours for histopathologic analysis. Lung sections were dehydrated with different concentrations of alcohol and then treated with paraffin embedding. The pathological changes of lung sections were assessed by using hematoxylin and eosin (H&E) staining.

### Enzyme-linked immunosorbent assay

After blood gas analyses, the remaining blood of abdominal aortic in rats was centrifuged, then the supernatants were taken for future use. The contents of cytokine and lung related index in serum were measured using. Levels of clam cell protein (CC16), surfactant protein-D (SP-D), interleukin (IL)-1β and IL-18 were confirmed by corresponding enzyme-linked immunosorbent assay (ELISA) kits (Yite Life Science R & D Co., LTD, Tianjin, China).

### Immunohistochemistry

The PPARγ and NF-κB expression were analysed by immunohistochemistry. Likewise, lung tissue samples were fixed, embedded, and then sliced into sections. The sections were incubated with primary antibodies of anti-PPARγ (1:300; CST, United States of America) and anti-NF-κB (1:500; CST, United States of America) overnight. On the second day, the slices were incubated with secondary antibodies, and finally reacted with diaminobenzidine (DAKO, Agilent Technologies Co., Copenhagen, Denmark). The analysis of the intensity of positive staining was performed through integrated optical density.

### Western blot

The primary antibodies used were as follows: ASC antibody (1:1,000, EL900169; Eterlife, Birmingham, United Kingdom), NLRP3 antibody (1:500, 15101; CST, Boston, United States of America), and Pro-caspase1 antibody (1:1,000, Ab179515; Abcam, Cambridge, United Kingdom). Glyceraldehyde-3-phosphate dehydrogenase (GAPDH) antibody (1:800, EL901118; Eterlife, Birmingham, United Kingdom) was a reference antibody. The secondary antibody concentration was 1:10,000.

Lung samples were lysed with a lysis buffer (Besseno Biotechnology Co., Tianjin, China). The protein quantity of lung tissue samples were assessed using the BCA method. The proteins were separated by SDS-PAGE and transferred onto polyvinylidene fluoride membranes. The membranes were first incubated with primary antibodies at 4°C overnight. On the following day, after incubation with the appropriate secondary antibody for 30 minutes, the protein bands were identified with an enhanced chemiluminescence solution. Alpha software processing system was used to check the optical density value of the target.

### Statistical analysis

All results were presented as mean ± standard deviation. The Shapiro-Wilk’s test was performed to analyse the normal distribution of data. The comparisons between the groups were checked by one-way analysis of variance, and *p* < 0.05 showed that the data have significant statistical difference. The statistics of the data were analyzed by Statistical Package for the Social Sciences 27 software.

## Results

### Berberine protect against lung injury induced by liver transplantation

**Figure 1:**
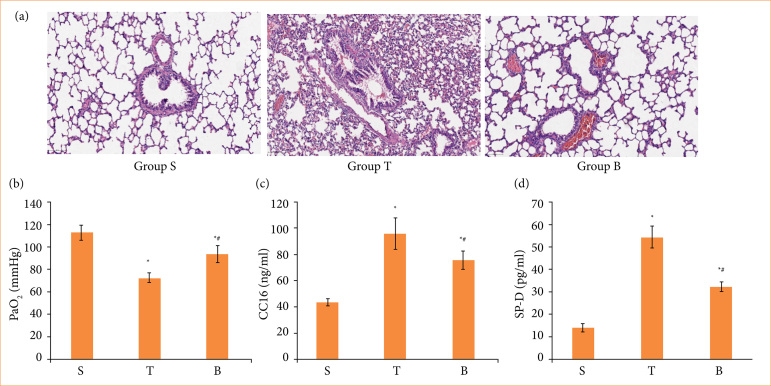
Berberine attenuated lung injury after liver transplantation. Sprague-Dawley rats were subjected to orthotopic autologous liver transplantation followed by 6 hours of reperfusion as indicated. Then, the lungs were collected for **(a)** hematoxylin and eosin staining, **(b)** arterial blood PaO_2_, **(c)** CC16 of serum , and **(d)** SP-D of serum. Error bars depict the standard deviations.

CC16: clam cell protein; SP-D: surfactant protein-D; PaO_2_: partial arterial oxygen pressure; *p < 0.05, as compared with group S; #*p* < 0.05, as compared with group T. Source: Elaborated by the authors.

Six hours after liver transplantation, no significant change was occurred in the lung tissue of the sham-operated group. However, lung tissue in T group was seriously damaged, mainly manifesting in pulmonary capillary congestion, obvious alveolar exudation, inflammatory cell infiltration, and alveolar septum thickening and edema. After BBR preconditioning, the pathological injuries of the animal samples caused by liver transplantation were significantly reduced (Fig. 1a). Figure 1b shows that the result of the Group T had a significant decrease in arterial oxygen partial pressure (PaO_2_), whereas PaO_2_ in the group S was in the normal range. The gas exchange capacity of lung tissue was greatly improved, after BBR pretreatment.

To address liver transplantation-induced acute lung injury, we examined the indicators of damage to the alveolar wall, such as CC16 and SP-D in serum by ELISA method. The concentrations of CC16 and SP-D were increased in the liver transplantation model (Figs. 1c and 1d). After the administration of BBR, the serum concentrations of SP-D and CC16 were obviously decreased compare with the T group (Figs. 1c and 1d).

### PPAR*γ*/NF-*κ*B pathway was involved in the mechanism of lung damage

**Figure 2:**
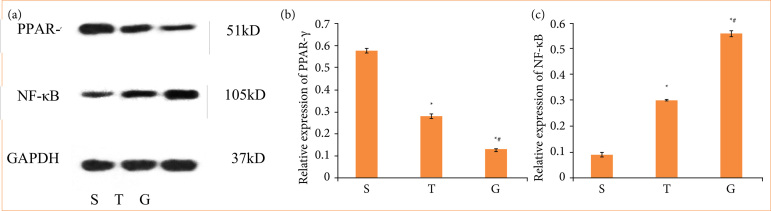
PPARγ/NF-κB signaling pathway was involved in the process of lung injury caused by liver transplantation. The proteins of PPAR-γ and NF-κB were analyzed by western blot analysis. **(a)** PPARγ and NF-κB, histogram showing the statistical comparison of **(b)** PPARγ and **(c)** NF-κB protein levels in each group.

To confirm the involvement of the PPARγ/NF-κB pathway in liver transplant-induced lung injury, we administered intraperitoneal injection of PPARγ antagonist GW9662 to rats. Results of Western blot manifested that the protein levels of PPARγ was downregulated, and the expression of NF-κB was up-regulated with the severity of lung injury in group T. The protein level of PPARγ was significantly decreased, and the expression of NF-κB was further up-regulated after GW9662 treatment (Fig. 2). These results suggested that inhibiting of PPARγ and activating of NF-κB signaling pathway may lead to the exacerbation of lung injury.

PPARγ: peroxisome proliferator-activated receptor gamma; NF-κB: nuclear factor-kappa B; *p < 0.05, compared with group S; #*p* < 0.05 compared with group T. Source: Elaborated by the authors.

### Berberine prevents lung injury by up-regulating peroxisome proliferator-activated receptor gamma protein expression and inhibiting the nuclear factor-kappa B protein expression

**Figure 3:**
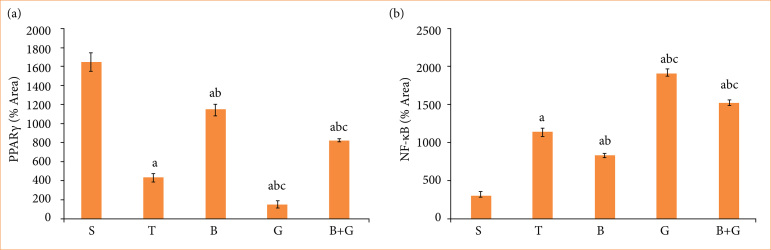
Berberine had a certain protective effect on lung injury through the PPARγ/NF-κB signaling pathway. **(a)** The level of PPARγ in lung tissue was measured by immunohistochemistry technique. **(b)** The level of NF-κB was measured by immunohistochemistry technique.

To verify the effect of BBR on the PPARγ/NF-κB pathway during lung injury, the GW9662 was added to the treatment group. We used immunohistochemical methods to evaluate the levels of PPARγ and NF-κB. As shown in Figs. 3a and 3b, the level of PPARγ was downregulated, and the level of NF-κB was upregulated in response to group T. However, after BBR pretreatment, the expression of PPARγ was activated, and the level of NF-κB was inhibited, and the administration of GW9662 attenuated the protective effects of BBR. These results indicated that BBR ameliorated lung injury induced by liver transplantation by up-regulating PPARγ expression level and inhibiting NF-κB signaling pathway.

PPARγ: peroxisome proliferator-activated receptor gamma; NF-κB: nuclear factor-kappa B; S: sham group; T: orthotopic autologous liver transplantation group; B: berberine pretreatment group; G: GW9662 pretreatment group; B+G: berberine + GW9662 group; ^a^ *p* < 0.05 compared with sham group; ^b^ *p* < 0.05 compared with group T; ^c^ *p* < 0.05 compared with group B. Source: Elaborated by the authors.

### Berberine suppressed NF-*κ*B-mediated cell pyroptosis pathway by activating the expression of PPAR*γ* in lung injury 

**Figure 4:**
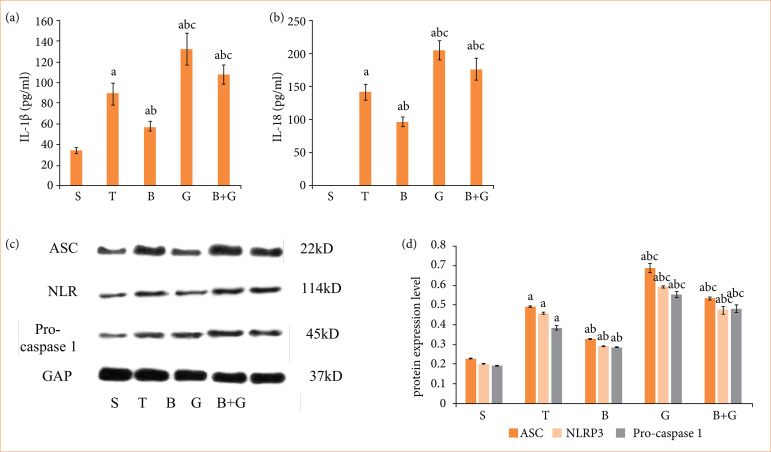
Berberine mitigated lung damage in rats undergoing liver transplantation through PPARγ/NF-κB-mediated cell pyroptosis signaling pathway. (**a** and **b**) Enzyme-linked immunosorbent assay measured the relative values of IL-1β and IL-18 concentration. (**c** and **d**) The expression of pyroptosis-associated proteins ASC, pro-caspase 1, and NLRP3 were detected by western blot analysis.

Pyroptosis is involved in the injury process of various organs. Therefore, we detected the proteins associated with pyroptosis in order to determine the protection mechanisms of BBR. The results of the ELISA are shown in Figs. 4a and 4b. The serum levels of IL-1β and IL-18 in group T were significantly upregulated. The serum levels of IL-1β and IL-18 in group B were significantly decreased compared with group T. After the administration of GW9662, the concentrations of IL-1β and IL-18 were increased compared to those in group B.

PPARγ: peroxisome proliferator-activated receptor gamma; NF-κB: nuclear factor-kappa B; IL: interleukin; S: sham group; T: orthotopic autologous liver transplantation group; B: berberine pretreatment group; G: GW9662 pretreatment group; B+G: berberine + GW9662 group; ASC: apoptosis-associated speck-like protein containing a CARD domain; NLRP3: nod-like receptor protein 3; ^a^ *p* < 0.05 compared with sham group; ^b^ *p* < 0.05 compared with group T; ^c^ *p* < 0.05 compared with group B. Source: Elaborated by the authors.

Western blot analysis exhibited that the expression of ASC, pro-caspase 1, and NLRP3 in group T were markedly upregulated. BBR administration significantly decreased the expression level of pyroptosis related proteins, and those effects were reversed by GW9662 (Figs. 4c and 4d). BBR preconditioning partially reduced the expression of cell pyroptosis during liver transplantation by activating the PPARγ protein and downregulating the NF-κB protein expression.

## Discussion

Pulmonary complications after liver transplantation lead to higher mortality and prolong the patient’s stay in hospital. Liver transplantation has great trauma and long-operation time. During the operation, the portal vein and inferior vena cava need to be blocked. When the new liver phase opens, gastrointestinal endotoxin, acid metabolites, and inflammatory mediators enter the systemic circulation, leading to acute lung injury. Our results indicated that the lung tissue structure were not abnormal in group S, while the pathological features of lung in group T were capillary congestion, obvious alveolar exudation, inflammatory cell infiltration, and alveolar septum thickening and edema. The degree of lung injury after BBR pretreatment was less than that of group T. These data indicated that liver transplantation induced lung injury.

CC16 mainly secreted by Clara cells, accounts for the highest content of protein in bronchoalveolar fluid and is a plasma biomarker that determines whether the pulmonary epithelial cells are intact. When the alveolar walls are damaged, CC16 enters the blood circulation in large quantities. Increasing findings have reported that CC16 can predict the development of lung disease caused by multiple factors, including COVID-19-associated lung injury, chronic obstructive pulmonary disease, asthma and idiopathic pulmonary fibrosis^14^-^17^. When lung barrier function is impaired, CC16 is easily detected in the blood.

A retrospective study found that sudden elevation of plasma CC16 was highly sensitive for evaluating if acute lung injury/acute respiratory distress syndrome had occurred^18^. The concentration level of CC16 in acute respiratory distress syndrome patients in intensive care unit was significantly increased, and the time of stay in intensive care unit was prolonged^19^. A study reported an increase in serum CC16 level before liver transplantation could predict the occurrence of acute respiratory distress syndrome after transplantation^20^. Plasma CC16 levels also predicted 90-day mortality in acute respiratory distress syndrome patients^21^. Significantly elevated CC16 levels were found in a lung-injury model of pig caused by multi-factor injuries, suggesting a good correlation between the CC16 level in plasma and lung injury^22^.

Surfactant protein (SP) is composed of the main components of the alveolar surface active substance. SP-D is one of the macro-molecular hydrophilic proteins, synthesizing and secreting from II alveolar epithelial cells into the alveolar cavities, when alveolar walls are damaged and permeability changes. Therefore, the elevated SP-D concentration in plasma was used as biomarker of lung injury severity^23^,^24^. The present study indicated that the concentration levels of CC16 and SP-D in group T were be markedly increased, compared to those in group S (*p* < 0.05), showing that lung tissue was damaged during liver transplantation and resulting in increased alveolar permeability.

We mainly research the associated mechanism by which BBR improves lung damage. Because of BBR upregulated the PPARγ protein expression in the adipose tissue^25^, we further examined whether BBR could alter PPARγ expression. PPARγ is a ligand-activated nuclear receptor, and the expression of genes such as immunity, inflammation, metabolism and cell differentiation is affected by activating PPARγ. Increasing studies have confirmed that the high expression of PPARγ may involve the protective mechanism of ischemia-reperfusion injury. Lu et al.^26^ showed that the mitochondrial-derived peptide improved postoperative acute lung injury caused by cardiac surgery through the PPARγ pathway. Propofol exhibited renoprotection on the ischemia-reperfusion injury by activating PPARγ/STAT3-dependent macrophage conversion^27^. Recent studies have reported that PPARγ transcription factors contributed to anti-inflammatory and antioxidant stress, in part by inhibiting NF-κB pathway activation^28^,^29^. Therefore, the protective mechanism of BBR against lung injury may associated with the modulation of PPARγ/NF-κB related pathway. We discovered that the expression level of PPARγ was downregulated in lung injury induced by liver transplantation, while BBR pretreatment increased PPARγ protein expression level in the current study. In addition, the expressions of NF-κB, ASC, NLRP3, and pro-caspase 1 were upregulated on lung injury induced by liver transplantation, which verified pyroptosis was activated in acute lung injury. We also provided evidence for the mechanism by examining the PPARγ/NF-κB-pyroptosis pathway.

Much evidence has shown that BBR has a certain protective capacity on lung injury induced by various causes. Studies have suggested that BBR pretreatment considerably mitigated lung injury and alleviated the degree of pulmonary edema and hypoxemia in septic mice^30^,^31^. Additionally, BBR alleviated influenza virus-induced pneumonia by suppressing NLRP3 inflammasome and reducing mitochondrial reactive oxygen species production^32^. A study also found that BBR had a certain anti-pulmonary fibrosis effect and inhibited inflammatory factors through the MAPK pathway in mice^33^.

In our current study, we administered BBR 200 mg/kg to verify its effects on lung injury after liver transplantation. Our results indicated that preoperative pretreatment of BBR could reduce the permeability of the alveolar wall, inhibit the occurrence of inflammatory response, and alleviate lung injury during liver transplantation. In this current study, BBR pretreatment decreased the expressions of NF-κB, ASC, pro-caspase 1, and NLRP3 and increased the protein expression of PPARγ.

In conclusion, our findings showed that BBR acts possibly by activating the PPARγ expression, inhibiting the NF-κB expression, and further inhibiting of pyroptosis signaling pathway.

## Conclusion

In our present experimental study, we examined the possible protective mechanism of BBR against lung injury caused by liver transplantation. Therefore, we think that BBR should be a useful therapeutic drug for improving lung injury. This result should be further verified in *in-vitro* models so as to better confirm the protective mechanisms of BBR against lung injury, which will be one of our future research directions.

## Data Availability

All data sets were generated or analyzed in the current study.
