# ERRATA: Berberine protects against lung injury induced by liver transplantation through upregulating PPARγ and suppressing NF-κB-mediated pyroptosis pathway

**DOI:** 10.1590/acb404925.errata

**Published:** 2025-10-24

In the manuscript “Berberine protects against lung injury induced by liver transplantation through upregulating PPARγ and suppressing NF-κB-mediated pyroptosis pathway”, which DOI is: https://doi.org/10.1590/acb404925, published in the journal Acta Cirúrgica Brasileira, 2025; v40; e404925, page 1, in the first affiliation:

Instead of

1. Tianjin Medical University – I Memorial Hospital-Department of Anesthesiology

Should be

1. Tianjin Medical University Chu Hsien-I Memorial Hospital – Department of Anesthesiology

